# Supplementary material for: Clinical evaluation of postoperative analgesia, cardiorespiratory parameters and changes in liver and renal function tests of paracetamol compared to meloxicam and carprofen in dogs undergoing ovariohysterectomy
Source: PLoS One. 2020 Feb 14;15(2):e0223697. doi: 10.1371/journal.pone.0223697 (PMC7021320; doi:10.1371/journal.pone.0223697)
Supplement: S1 Appendix — (DOCX) [file pone.0223697.s001.docx]

**Appendix 1. The University of Melbourne’s Pain Scale (UMPS) and the Dynamic Interactive Visual Analog Scale (DIVAS)** [37,38,51]**.**

| The results of the patient’s evaluation was interpreted as follows:  Category | Description of pain  1 – 5 = mild pain  6 – 13 = moderate pain  14 – 21 = severe pain  21–27= unbearable pain | | Score |  |
| --- | --- | --- | --- | --- |
| 1. Physiological parameters | 1. Physiological data within the reference range | | 0 |  |
|  | 1. Dilated pupils | | 2 |  |
|  | 1. Increase in heart rate (HR) relative to baseline:   >20 %  >50%  >100% | | 1  2  3 |  |
|  | 1. Increase in respiratory rate (RR) relative to baseline:   >20%  >50%  >100% | | 1  2  3 |  |
|  | 1. Hyperthermia | | 1 |  |
|  | 1. Salivation | | 2 |  |
| 1. Response to palpation | 1. No behavioural changes 2. Protective reactions* / when touched 3. Protective reactions* / before touching it is protected | | 0  2  3 |  |
| 1. Activity | 1. At rest: sleeping 2. At rest: semi-conscious 3. At rest: awake 4. Eating 5. Restless (walk constantly, gets up and back to bed) 6. Sudden and frequent movements (wallowing and hitting) | | 0  0  1  0  2  3 |  |
| 1. State of mind | 1. Submissive 2. Anxious 3. Fearful 4. Aggressive | | 0  1  2  3 |  |
| 1. Posture | 1. Protects the affected area (foetal position) 2. Lateral decubitus 3. Sternal decubitus   Choose one:   1. Sitting or standing 2. Moving 3. Abnormal posture | | 2  0  1  2  1  2 |  |
| 1. Vocalisation + | 1. Does not vocalise 2. Vocalises when touched 3. Intermittent vocalisation 4. Continuous vocalisation | | 0  2  2  3 |  |
| * Protective reactions include movements of the head to the affected area, licking, biting, scratching the wound, tensing muscles, and protective posturing.  + Does not include warning barks. | |  | | |

| DIVAS include the observation of the patient at a certain distance | **Evaluation criteria** | |
| --- | --- | --- |
|  | DIVAS I (distance observation) | Vocalisation, posture changes, facial expression, blepharospasm, salivation, wound licking and breathing type |
|  | DIVAS II (the evaluator must approach the animal and interact) | Appetite, motor capacity and behaviour |
|  | DIVAS III (response to palpation) | Palpation of the area of ​​the wound to assess body tension at the time of exerting pressure on adjacent areas |

Evaluation criteria that should consider during the use of Interactive Visual Analog Scale (DIVAS).
