# Supplementary material for: Clinical evaluation of postoperative analgesia, cardiorespiratory parameters and changes in liver and renal function tests of paracetamol compared to meloxicam and carprofen in dogs undergoing ovariohysterectomy
Source: PLoS One. 2020 Feb 14;15(2):e0223697. doi: 10.1371/journal.pone.0223697 (PMC7021320; doi:10.1371/journal.pone.0223697)
Supplement: S1 Table — (DOCX) [file pone.0223697.s002.docx]

S1 Table 1. Cardiorespiratory variables (HR, MAP, SpO2, ETCO2), temperature and ET_ISO_ in each individual during the intraoperative in the different study groups.

**Heart rate (beats per minute)**

| Carprofeno | Meloxicam | Paracetamol |
| --- | --- | --- |
| 147 | 108 | 103 |
| 80 | 125 | 92 |
| 80 | 124 | 86 |
| 130 | 130 | 105 |
| 130 | 131 | 75 |
| 92 | 126 | 74 |
| 92 | 86 | 70 |
| 92 | 120 | 104 |
| 81 | 102 | 108 |
| 88 | 84 | 145 |
| 111 | 82 | 159 |
| 112 | 83 | 140 |
| 129 | 84 | 127 |
| 100 | 90 | 123 |
| 101 | 88 | 95 |
| 97 | 92 | 111 |
| 86 | 95 | 139 |
| 101 | 80 | 145 |
| 96 | 82 | 118 |
| 114 | 85 | 95 |
| 104 | 99 | 89 |
| 84 | 100 | 86 |
| 85 | 109 | 108 |
| 83 | 141 | 111 |
| 87 | 100 | 130 |
| 77 | 90 | 95 |
| 85 | 81 | 89 |
| 98 | 109 | 92 |
| 105 | 111 | 105 |
| 79 | 117 | 140 |
| 72 | 85 | 160 |
| 73 | 84 | 166 |
| 88 | 81 | 170 |
| 95 | 79 | 159 |
| 107 | 101 | 158 |
| 92 | 100 | 160 |
| 93 | 103 | 156 |
| 98 | 98 | 148 |
| 95 | 93 | 154 |
| 120 | 91 | 145 |
| 123 | 93 | 128 |
| 125 | 111 | 117 |
| 119 | 97 | 121 |
| 118 | 111 | 110 |
| 117 | 100 | 78 |
| 123 | 104 | 75 |
| 97 | 93 | 52 |
| 88 | 90 | 53 |
| 91 | 125 | 85 |
| 97 | 112 | 96 |
| 77 | 102 | 110 |
| 77 | 101 | 109 |
| 79 | 95 | 113 |
| 138 | 106 | 115 |
| 128 | 128 | 119 |
| 141 | 123 | 114 |
| 153 | 124 | 118 |
| 159 | 130 | 83 |
| 148 | 127 | 82 |
| 150 | 126 | 81 |
| 119 | 122 | 80 |
| 122 | 121 | 81 |
| 117 | 148 | 78 |
| 109 | 147 | 77 |
| 91 | 146 | 89 |
| 90 | 145 | 77 |
| 103 | 142 | 83 |
|  | 126 | 93 |
|  | 122 | 86 |
|  |  | 86 |
|  |  | 95 |

**Heart rate (beats per minute)**

| **Descriptive statistics** | **Carprofeno** | **Meloxicam** | **Paracetamol** |
| --- | --- | --- | --- |
| Number of values | 67 | 69 | 71 |
|  |  |  |  |
| Minimum | 72 | 79 | 52 |
| 25% percentile | 88 | 90.5 | 86 |
| Median | 98 | 102 | 105 |
| 75% percentile | 119 | 124 | 128 |
| Maximum | 159 | 148 | 170 |
|  |  |  |  |
| Mean | 104.1 | 107.0 | 108.7 |
| Std deviation | 21.72 | 19.37 | 29.18 |
| Std error of mean | 2.654 | 2.332 | 3.463 |
|  |  |  |  |
| Lower 95% CI | 98.85 | 102.4 | 101.8 |
| Upper 95% CI | 109.4 | 111.7 | 115.6 |

**Median Arterial blood Pressure (mmHg)**

| Carprofeno | Meloxicam | Paracetamol |
| --- | --- | --- |
| 77 | 90 | 86 |
| 70 | 91 | 86 |
| 74 | 86 | 86 |
| 95 | 92 | 81 |
| 85 | 94 | 96 |
| 85 | 92 | 92 |
| 79 | 58 | 86 |
| 79 | 42 | 93 |
| 70 | 81 | 96 |
| 75 | 70 | 103 |
| 70 | 50 | 98 |
| 66 | 53 | 100 |
| 64 | 49 | 80 |
| 79 | 74 | 76 |
| 100 | 65 | 92 |
| 103 | 85 | 106 |
| 98 | 83 | 101 |
| 87 | 72 | 90 |
| 95 | 71 | 83 |
| 61 | 74 | 85 |
| 93 | 58 | 50 |
| 76 | 59 | 65 |
| 79 | 71 | 102 |
| 93 | 108 | 98 |
| 85 | 95 | 80 |
| 93 | 85 | 78 |
| 64 | 84 | 70 |
| 72 | 71 | 48 |
| 58 | 91 | 54 |
| 83 | 105 | 100 |
| 50 | 97 | 87 |
| 44 | 87 | 89 |
| 57 | 70 | 64 |
| 71 | 71 | 65 |
| 36 | 62 | 86 |
| 39 | 71 | 91 |
| 44 | 80 | 109 |
| 51 | 69 | 96 |
| 58 | 64 | 105 |
| 65 | 64 | 99 |
| 70 | 62 | 89 |
| 73 | 75 | 81 |
| 77 | 75 | 76 |
| 73 | 92 | 94 |
| 66 | 94 | 127 |
| 70 | 85 | 126 |
| 71 | 85 | 67 |
| 58 | 82 | 75 |
| 57 | 77 | 75 |
| 57 | 85 | 78 |
| 91 | 92 | 74 |
| 73 | 95 | 85 |
| 57 | 86 | 85 |
| 57 | 80 | 82 |
| 70 | 76 | 70 |
| 73 | 87 | 53 |
| 77 | 88 | 50 |
| 73 | 105 | 65 |
| 66 | 112 | 90 |
| 70 | 116 | 90 |
| 71 | 103 | 72 |
| 65 | 100 | 77 |
| 69 | 71 | 76 |
| 67 | 72 | 78 |
| 59 | 102 | 89 |
| 64 | 113 | 84 |
| 67 | 120 | 77 |
| 76 | 91 | 102 |
|  | 88 | 90 |

**Median Arterial blood Pressure (mmHg)**

| **Descriptive statistics** | **Carprofeno** | **Meloxicam** | **Paracetamol** |
| --- | --- | --- | --- |
| Number of values | 68 | 69 | 69 |
|  |  |  |  |
| Minimum | 36 | 42 | 48 |
| 25% percentile | 64 | 71 | 76 |
| Median | 70.50 | 84 | 86 |
| 75% percentile | 79 | 92 | 95 |
| Maximum | 103 | 120 | 127 |
|  |  |  |  |
| Mean | 71.18 | 81.78 | 84.48 |
| Std deviation | 14.28 | 16.61 | 15.86 |
| Std error of mean | 1.732 | 2.000 | 1.909 |
|  |  |  |  |
| Lower 95% CI | 67.72 | 77.79 | 80.67 |
| Upper 95% CI | 74.63 | 85.77 | 88.29 |

**Temperature (ºC)**

| Carprofeno | Meloxicam | Paracetamol |
| --- | --- | --- |
| 36.4 | 35.9 | 37 |
| 36.4 | 36.1 | 36.3 |
| 36.2 | 36.1 | 36.4 |
| 36.1 | 36.1 | 36.4 |
| 36 | 36.1 | 36.4 |
| 36 | 36.3 | 36.4 |
| 36 | 35.8 | 36.4 |
| 35 | 35.5 | 35.4 |
| 36 | 35.7 | 35.4 |
| 35.6 | 35.7 | 35.1 |
| 35 | 35.7 | 35.2 |
| 35 | 35.7 | 35.3 |
| 34.9 | 35.8 | 35.3 |
| 34.7 | 36.2 | 35.3 |
| 34.7 | 36.2 | 37.1 |
| 34.6 | 36.1 | 37.1 |
| 34.6 | 36 | 37.1 |
| 35.9 | 36 | 37.1 |
| 35.7 | 36 | 37.1 |
| 35.5 | 36 | 37.2 |
| 34.7 | 34.4 | 37.2 |
| 34.5 | 34.4 | 37.2 |
| 35.7 | 34.3 | 36.5 |
| 35.7 | 34.4 | 36.5 |
| 35.6 | 33.9 | 36.5 |
| 35.9 | 33.9 | 36.5 |
| 35.8 | 33.9 | 36.7 |
| 35.7 | 37.2 | 36.9 |
| 35.7 | 37.1 | 39.1 |
| 35.7 | 37.2 | 37 |
| 35.6 | 37 | 37 |
| 35.6 | 36.7 | 36.9 |
| 36 | 36.6 | 36.8 |
| 36 | 36.6 | 36.8 |
| 36 | 35.9 | 36.8 |
| 35.9 | 35.9 | 36.8 |
| 35.9 | 35.8 | 34.9 |
| 35.7 | 35.7 | 34.7 |
| 35.7 | 35.7 | 34.6 |
| 36.2 | 35.7 | 34.4 |
| 36.2 | 35.6 | 34.3 |
| 36.1 | 36.1 | 34.2 |
| 36.1 | 36.1 | 34 |
| 35.9 | 36.1 | 36.4 |
| 35.9 | 36.1 | 36.3 |
| 35.2 | 36.1 | 36.1 |
| 37.2 | 36 | 36.1 |
| 37.2 | 35.9 | 36 |
| 37 | 34.1 | 36.1 |
| 36.8 | 34.1 | 36 |
| 36.8 | 34.2 | 36.3 |
| 36.7 | 34.2 | 36.3 |
| 36.6 | 34.2 | 36.3 |
| 36.4 | 34.2 | 36.3 |
| 36.2 | 34.2 | 36.2 |
| 36.2 | 37.1 | 36.2 |
| 36.1 | 37.1 | 36.1 |
| 36.1 | 37 | 36 |
| 36.1 | 37 | 35.9 |
| 36 | 36.9 | 35.9 |
| 36.5 | 36.9 | 35.9 |
| 36.5 | 36.8 | 36 |
| 36.5 | 37.2 | 35.9 |
| 36.4 | 37.2 | 35.8 |
| 36.4 | 37.2 | 37.7 |
| 36.4 | 37.1 | 37.7 |
| 36.4 | 37.1 | 37.7 |
|  | 37 | 37.6 |
|  | 36.9 | 37.4 |
|  |  | 37.4 |
|  |  | 37.3 |

**Temperature (ºC)**

| **Descriptive statistics** | **Carprofeno** | **Meloxicam** | **Paracetamol** |
| --- | --- | --- | --- |
| Number of values | 67 | 69 | 71 |
|  |  |  |  |
| Minimum | 34.50 | 33.90 | 34.00 |
| 25% percentile | 35.70 | 35.70 | 35.90 |
| Median | 36.00 | 36.00 | 36.40 |
| 75% percentile | 36.40 | 36.85 | 37.00 |
| Maximum | 37.20 | 37.20 | 39.10 |
|  |  |  |  |
| Mean | 35.91 | 35.90 | 36.31 |
| Std deviation | 0.6199 | 1.011 | 0.9369 |
| Std error of mean | 0.07574 | 0.1217 | 0.1112 |
|  |  |  |  |
| Lower 95% CI | 35.76 | 35.66 | 36.09 |
| Upper 95% CI | 36.06 | 36.14 | 36.53 |

**SpO_2_ (%)**

| Carprofeno | Meloxicam | Paracetamol |
| --- | --- | --- |
| 98 | 97 | 95 |
| 100 | 98 | 96 |
| 100 | 97 | 95 |
| 98 | 98 | 96 |
| 98 | 99 | 96 |
| 99 | 98 | 95 |
| 98 | 95 | 95 |
| 97 | 95 | 98 |
| 99 | 98 | 98 |
| 98 | 99 | 98 |
| 99 | 98 | 98 |
| 99 | 98 | 98 |
| 99 | 98 | 98 |
| 99 | 99 | 98 |
| 98 | 98 | 99 |
| 98 | 98 | 98 |
| 98 | 98 | 97 |
| 98 | 99 | 99 |
| 98 | 98 | 98 |
| 98 | 98 | 97 |
| 98 | 99 | 98 |
| 98 | 99 | 98 |
| 98 | 98 | 97 |
| 98 | 99 | 96 |
| 98 | 99 | 97 |
| 98 | 99 | 97 |
| 99 | 99 | 97 |
| 99 | 98 | 96 |
| 98 | 98 | 96 |
| 98 | 98 | 99 |
| 98 | 98 | 99 |
| 98 | 98 | 99 |
| 98 | 98 | 99 |
| 99 | 98 | 99 |
| 97 | 98 | 98 |
| 99 | 98 | 98 |
| 100 | 98 | 98 |
| 100 | 97 | 97 |
| 100 | 98 | 98 |
| 99 | 98 | 99 |
| 99 | 98 | 99 |
| 98 | 98 | 98 |
| 98 | 98 | 98 |
| 97 | 98 | 98 |
| 99 | 98 | 98 |
| 97 | 99 | 98 |
| 97 | 96 | 98 |
| 99 | 97 | 96 |
| 99 | 98 | 97 |
| 99 | 98 | 98 |
| 99 | 98 | 98 |
| 99 | 99 | 98 |
| 99 | 98 | 96 |
| 99 | 98 | 95 |
| 96 | 98 | 96 |
| 98 | 98 | 95 |
| 96 | 98 | 96 |
| 95 | 98 | 99 |
| 95 | 97 | 99 |
| 94 | 97 | 99 |
| 94 | 97 | 99 |
| 95 | 97 | 100 |
| 95 | 99 | 96 |
| 100 | 99 | 97 |
| 99 | 99 | 97 |
| 90 | 99 | 97 |
| 91 | 99 | 97 |
| 97 | 99 | 96 |
|  | 99 | 95 |
|  |  | 97 |
|  |  | 98 |

**SpO_2_**

| **Descriptive statistics** | **Carprofeno** | **Meloxicam** | **Paracetamol** |
| --- | --- | --- | --- |
| Number of values | 68 | 69 | 71 |
|  |  |  |  |
| Minimum | 90 | 95 | 95 |
| 25% percentile | 98 | 98 | 96 |
| Median | 98 | 98 | 98 |
| 75% percentile | 99 | 99 | 98 |
| Maximum | 100 | 99 | 100 |
|  |  |  |  |
| Mean | 97.82 | 98.04 | 97.39 |
| Std deviation | 1.884 | 0.8477 | 1.282 |
| Std error of mean | 0.2285 | 0.1021 | 0.1521 |
|  |  |  |  |
| Lower 95% CI | 97.37 | 97.84 | 97.09 |
| Upper 95% CI | 98.28 | 98.25 | 97.70 |

**EtCO_2_ (mmHg)**

| Carprofeno | Meloxicam | Paracetamol |
| --- | --- | --- |
| 37 | 36 | 43 |
| 40 | 37 | 43 |
| 40 | 36 | 47 |
| 37 | 33 | 41 |
| 37 | 33 | 35 |
| 32 | 35 | 33 |
| 32 | 38 | 32 |
| 36 | 30 | 37 |
| 36 | 35 | 36 |
| 38 | 36 | 35 |
| 38 | 40 | 40 |
| 30 | 40 | 42 |
| 29 | 40 | 43 |
| 28 | 38 | 43 |
| 26 | 36 | 37 |
| 26 | 35 | 33 |
| 30 | 38 | 34 |
| 33 | 40 | 38 |
| 27 | 40 | 36 |
| 30 | 38 | 35 |
| 29 | 33 | 35 |
| 30 | 34 | 35 |
| 37 | 34 | 35 |
| 37 | 31 | 35 |
| 40 | 32 | 36 |
| 34 | 33 | 35 |
| 31 | 34 | 40 |
| 31 | 31 | 43 |
| 34 | 31 | 40 |
| 34 | 32 | 39 |
| 36 | 30 | 44 |
| 32 | 31 | 42 |
| 36 | 33 | 44 |
| 30 | 33 | 45 |
| 37 | 35 | 46 |
| 35 | 35 | 46 |
| 30 | 33 | 30 |
| 27 | 34 | 33 |
| 29 | 35 | 30 |
| 32 | 35 | 37 |
| 34 | 36 | 35 |
| 34 | 32 | 34 |
| 35 | 33 | 31 |
| 35 | 32 | 35 |
| 35 | 31 | 31 |
| 34 | 31 | 33 |
| 38 | 34 | 30 |
| 35 | 34 | 31 |
| 35 | 30 | 43 |
| 37 | 28 | 42 |
| 35 | 25 | 36 |
| 33 | 25 | 38 |
| 33 | 26 | 38 |
| 33 | 26 | 38 |
| 32 | 31 | 39 |
| 42 | 39 | 39 |
| 43 | 39 | 40 |
| 37 | 36 | 37 |
| 50 | 34 | 36 |
| 43 | 33 | 36 |
| 36 | 33 | 38 |
| 36 | 34 | 33 |
| 35 | 32 | 33 |
| 37 | 30 | 35 |
| 37 | 31 | 32 |
| 38 | 30 | 32 |
| 38 | 30 | 32 |
| 38 | 32 | 35 |
|  | 33 | 38 |
|  |  | 34 |
|  |  | 31 |

**EtCO_2_ mmHg**

| **Descriptive statistics** | **Carprofeno** | **Meloxicam** | **Paracetamol** |
| --- | --- | --- | --- |
| Number of values | 68 | 69 | 71 |
|  |  |  |  |
| Minimum | 26 | 25 | 30 |
| 25% percentile | 32 | 31 | 34 |
| Median | 35 | 33 | 36 |
| 75% percentile | 37 | 36 | 40 |
| Maximum | 50 | 40 | 47 |
|  |  |  |  |
| Mean | 34.57 | 33.52 | 37.01 |
| Std deviation | 4.338 | 3.530 | 4.396 |
| Std error of mean | 0.5260 | 0.4249 | 0.5218 |
|  |  |  |  |
| Lower 95% CI | 33.52 | 32.67 | 35.97 |
| Upper 95% CI | 35.62 | 34.37 | 38.05 |

**Et_ISO_**

| Carprofeno | Meloxicam | Paracetamol |
| --- | --- | --- |
| 0.79 | 0.90 | 1.09 |
| 1.58 | 1.29 | 0.80 |
| 0.80 | 1.03 | 0.90 |
| 1.59 | 1.70 | 1.02 |
| 0.77 | 1.04 | 0.95 |
| 1.66 | 0.93 | 1.25 |
| 0.82 | 1.03 | 1.25 |
| 1.54 | 0.77 | 1.33 |
| 1.63 | 1.23 | 1.15 |
| 0.83 | 1.36 | 1.21 |
| 0.79 | 1.11 | 1.21 |
| 0.80 | 0.95 | 1.02 |
| 1.50 | 1.06 | 1.24 |
| 1.56 | 1.01 | 1.04 |
| 0.80 | 1.65 | 1.22 |
| 0.82 | 1.18 | 0.97 |
| 1.60 | 0.94 | 1.04 |
| 1.51 | 1.03 | 0.92 |
| 1.58 | 1.18 | 1.08 |
| 0.80 | 1.07 | 0.98 |
| 1.57 | 0.89 | 1.18 |
| 1.52 | 0.95 | 1.17 |
| 1.55 | 0.85 | 1.21 |
| 1.62 | 1.25 | 0.91 |
| 1.56 | 1.64 | 0.99 |
| 1.65 | 1.10 | 0.93 |
| 0.79 | 1.00 | 1.20 |
| 1.58 | 1.21 | 1.12 |
| 0.80 | 0.74 | 0.93 |
| 1.66 | 1.03 | 0.90 |
| 0.81 | 1.02 | 1.05 |
| 0.76 | 0.92 | 1.03 |
| 1.65 | 1.28 | 0.95 |
| 1.58 | 0.78 | 0.97 |
| 1.63 | 1.35 | 0.92 |
| 0.79 | 1.24 | 1.07 |
| 0.75 | 1.31 | 1.14 |
| 1.57 | 0.84 | 1.27 |
| 0.82 | 1.33 | 1.19 |
| 1.60 | 1.58 | 1.21 |
| 0.76 | 0.90 | 0.96 |
| 0.81 | 1.03 | 1.00 |
| 0.78 | 1.35 | 1.06 |
| 1.45 | 1.32 | 0.89 |
| 0.77 | 0.94 | 1.05 |
| 1.62 | 1.39 | 1.08 |
| 1.53 | 1.23 | 0.97 |
| 1.61 | 1.12 | 1.08 |
| 0.75 | 0.72 | 1.21 |
| 0.79 | 1.03 | 1.18 |
| 1.58 | 0.90 | 1.29 |
| 1.51 | 0.85 | 1.37 |
| 0.82 | 1.13 | 1.10 |
| 0.77 | 1.01 | 1.02 |
| 1.54 | 0.92 | 1.00 |
| 1.51 | 1.37 | 1.38 |
| 0.81 | 1.22 | 0.90 |
| 1.56 | 1.24 | 1.21 |
| 1.57 | 1.38 | 1.03 |
| 0.79 | 1.08 | 0.96 |
| 0.81 | 1.52 | 1.25 |
| 1.49 | 0.98 | 1.22 |
| 1.60 | 1.14 | 0.87 |
| 0.72 | 1.00 | 1.25 |
| 1.61 | 1.36 | 1.00 |
| 0.78 | 1.31 | 0.89 |
| 0.82 | 1.47 | 0.93 |
|  | 0.83 | 1.13 |
|  | 0.92 | 0.91 |
|  |  | 1.16 |
|  |  | 1.19 |

**EtIso**

| **Descriptive statistics** | **Carprofeno** | **Meloxicam** | **Paracetamol** |
| --- | --- | --- | --- |
| Number of values | 67 | 69 | 71 |
|  |  |  |  |
| Minimum | 0.7200 | 0.7200 | 0.8000 |
| 25% percentile | 0.8000 | 0.9400 | 0.9600 |
| Median | 1.500 | 1.070 | 1.060 |
| 75% percentile | 1.580 | 1.300 | 1.210 |
| Maximum | 1.660 | 1.700 | 1.380 |
|  |  |  |  |
| Mean | 1.212 | 1.122 | 1.078 |
| Std deviation | 0.3956 | 0.2296 | 0.1368 |
| Std error of mean | 0.04833 | 0.02764 | 0.01624 |
|  |  |  |  |
| Lower 95% CI | 1.115 | 1.067 | 1.046 |
| Upper 95% CI | 1.308 | 1.177 | 1.111 |
