# Supplementary material for: Clinical evaluation of postoperative analgesia, cardiorespiratory parameters and changes in liver and renal function tests of paracetamol compared to meloxicam and carprofen in dogs undergoing ovariohysterectomy
Source: PLoS One. 2020 Feb 14;15(2):e0223697. doi: 10.1371/journal.pone.0223697 (PMC7021320; doi:10.1371/journal.pone.0223697)
Supplement: S2 Table — (DOCX) [file pone.0223697.s003.docx]

S2 Table 2. Serum biochemical levels of ALT, AST, ALP, Direct bilirubin, Indirect bilirubin, Total bilirubin, Urea, Creatinine, Albumin and Glucose in each individual during postoperative in the different study groups.

|  | Carprofeno | |  | Meloxicam | |  | Paracetamol | |  |
| --- | --- | --- | --- | --- | --- | --- | --- | --- | --- |
| **ALT** | T0 | T48 | T96 | T0 | T48 | T96 | T0 | T48 | T96 |
|  | 62 | 72 | 73 | 68 | 72 | 74 | 69 | 76 | 79 |
|  | 66 | 74 | 76 | 67 | 75 | 73 | 72 | 79 | 96 |
|  | 75 | 79 | 80 | 67 | 69 | 74 | 74 | 68 | 97 |
|  | 70 | 76 | 75 | 74 | 76 | 80 | 62 | 68 | 66 |
|  | 69 | 76 | 78 | 65 | 68 | 71 | 56 | 68 | 87 |
|  | 63 | 60 | 60 | 66 | 89 | 89 | 68 | 72 | 76 |
|  | 68 | 80 | 86 | 63 | 68 | 68 | 65 | 68 | 79 |
|  | 62 | 76 | 79 | 65 | 74 | 74 | 62 | 70 | 66 |
|  | 62 | 68 | 76 | 68 | 68 | 68 | 64 | 70 | 72 |
|  | 69 | 76 | 76 | 68 | 72 | 72 | 68 | 79 | 82 |

**ALT**

| Descriptive statistics | Carprofeno | | | Meloxicam | | | Paracetamol | | |
| --- | --- | --- | --- | --- | --- | --- | --- | --- | --- |
|  | T0 | T48 | T96 | T0 | T48 | T96 | T0 | T48 | T96 |
| Number of values | 10 | 10 | 10 | 10 | 10 | 10 | 10 | 10 | 10 |
|  |  |  |  |  |  |  |  |  |  |
| Minimum | 62 | 60 | 60 | 63 | 68 | 68 | 56 | 68 | 66 |
| 25% percentile | 62 | 71 | 74.50 | 65 | 68 | 70.25 | 62 | 68 | 70.50 |
| Median | 67 | 76 | 76 | 67 | 72 | 73.50 | 66.50 | 70 | 79 |
| 75% percentile | 69.25 | 76.75 | 79.25 | 68 | 75.25 | 75.50 | 69.75 | 76.75 | 89.25 |
| Maximum | 75 | 80 | 86 | 74 | 89 | 89 | 74 | 79 | 97 |
|  |  |  |  |  |  |  |  |  |  |
| Mean | 66.60 | 73.70 | 75.90 | 67.10 | 73.10 | 74.30 | 66 | 71.80 | 80 |
| Std deviation | 4.377 | 5.889 | 6.624 | 2.923 | 6.350 | 6.201 | 5.312 | 4.541 | 10.91 |
| Std error of mean | 1.384 | 1.862 | 2.095 | 0.9244 | 2.008 | 1.961 | 1.680 | 1.436 | 3.451 |
|  |  |  |  |  |  |  |  |  |  |
| Lower 95% CI | 63.47 | 69.49 | 71.16 | 65.01 | 68.56 | 69.86 | 62.20 | 68.55 | 72.19 |
| Upper 95% CI | 69.73 | 77.91 | 80.64 | 69.19 | 77.64 | 78.74 | 69.80 | 75.05 | 87.81 |

| **AST** | Carprofeno | |  | Meloxicam | |  | Paracetamol | |  |
| --- | --- | --- | --- | --- | --- | --- | --- | --- | --- |
|  | T0 | T48 | T96 | T0 | T48 | T96 | T0 | T48 | T96 |
|  | 35 | 48 | 53 | 57 | 48 | 51 | 67 | 87 | 89 |
|  | 46 | 58 | 60 | 52 | 62 | 62 | 65 | 80 | 97 |
|  | 73 | 78 | 81 | 53 | 50 | 55 | 66 | 70 | 54 |
|  | 69 | 72 | 74 | 68 | 70 | 72 | 57 | 59 | 57 |
|  | 68 | 74 | 75 | 63 | 66 | 69 | 45 | 57 | 46 |
|  | 68 | 65 | 65 | 68 | 74 | 74 | 47 | 50 | 54 |
|  | 62 | 72 | 77 | 58 | 70 | 70 | 74 | 68 | 76 |
|  | 64 | 65 | 65 | 64 | 68 | 68 | 64 | 68 | 65 |
|  | 61 | 74 | 80 | 65 | 70 | 70 | 68 | 72 | 89 |
|  | 70 | 74 | 74 | 56 | 61 | 61 | 64 | 74 | 72 |

**AST**

| Descriptive statistics | Carprofeno | | | Meloxicam | | | Paracetamol | | |
| --- | --- | --- | --- | --- | --- | --- | --- | --- | --- |
|  | T0 | T48 | T96 | T0 | T48 | T96 | T0 | T48 | T96 |
| Number of values | 10 | 10 | 10 | 10 | 10 | 10 | 10 | 10 | 10 |
|  |  |  |  |  |  |  |  |  |  |
| Minimum | 35 | 48 | 53 | 52 | 48 | 51 | 45 | 50 | 46 |
| 25% percentile | 57.25 | 63.25 | 63.75 | 55.25 | 58.25 | 59.50 | 54.50 | 58.50 | 54 |
| Median | 66 | 72 | 74 | 60.50 | 67 | 68.50 | 64.50 | 69 | 68.50 |
| 75% percentile | 69.25 | 74 | 77.75 | 65.75 | 70 | 70.50 | 67.25 | 75.50 | 89 |
| Maximum | 73 | 78 | 81 | 68 | 74 | 74 | 74 | 87 | 97 |
|  |  |  |  |  |  |  |  |  |  |
| Mean | 61.60 | 68 | 70.40 | 60.40 | 63.90 | 65.20 | 61.70 | 68.50 | 69.90 |
| Std deviation | 11.99 | 9.177 | 9.216 | 5.948 | 8.774 | 7.642 | 9.286 | 10.98 | 17.55 |
| Std error of mean | 3.792 | 2.902 | 2.914 | 1.881 | 2.775 | 2.417 | 2.937 | 3.471 | 5.551 |
|  |  |  |  |  |  |  |  |  |  |
| Lower 95% CI | 53.02 | 61.43 | 63.81 | 56.15 | 57.62 | 59.73 | 55.06 | 60.65 | 57.34 |
| Upper 95% CI | 70.18 | 74.57 | 76.99 | 64.65 | 70.18 | 70.67 | 68.34 | 76.35 | 82.46 |

| **ALP** | Carprofeno | |  | Meloxicam | |  | Paracetamol | |  |
| --- | --- | --- | --- | --- | --- | --- | --- | --- | --- |
|  | T0 | T48 | T96 | T0 | T48 | T96 | T0 | T48 | T96 |
|  | 84 | 156 | 90 | 147 | 170 | 160 | 100 | 170 | 166 |
|  | 145 | 180 | 125 | 90 | 70 | 78 | 70 | 96 | 120 |
|  | 145 | 164 | 148 | 67 | 73 | 75 | 58 | 96 | 131.8 |
|  | 74 | 71 | 70 | 123 | 110 | 102 | 95 | 90 | 85 |
|  | 67 | 77 | 85 | 87 | 74 | 77 | 70 | 87 | 95 |
|  | 62 | 73 | 73 | 70 | 98 | 98 | 110 | 100 | 110 |
|  | 65 | 65 | 108 | 62 | 96 | 96 | 87 | 87 | 90 |
|  | 72 | 80 | 84 | 74 | 98 | 98 | 65 | 74 | 64 |
|  | 89 | 86 | 86 | 70 | 78 | 78 | 62 | 69 | 79 |
|  | 70 | 90 | 90 | 74 | 86 | 86 | 75 | 80 | 94 |

**ALP**

| Descriptive statistics | Carprofeno | | | Meloxicam | | | Paracetamol | | |
| --- | --- | --- | --- | --- | --- | --- | --- | --- | --- |
|  | T0 | T48 | T96 | T0 | T48 | T96 | T0 | T48 | T96 |
| Number of values | 10 | 10 | 10 | 10 | 10 | 10 | 10 | 10 | 10 |
|  |  |  |  |  |  |  |  |  |  |
| Minimum | 62 | 65 | 70 | 62 | 70 | 75 | 58 | 69 | 64 |
| 25% percentile | 66.50 | 72.50 | 81.25 | 69.25 | 73.75 | 77.75 | 64.25 | 78.50 | 83.50 |
| Median | 73 | 83 | 88 | 74 | 91 | 91 | 72.50 | 88.50 | 94.50 |
| 75% percentile | 103 | 158 | 112.3 | 98.25 | 101 | 99 | 96.25 | 97 | 123 |
| Maximum | 145 | 180 | 148 | 147 | 170 | 160 | 110 | 170 | 166 |
|  |  |  |  |  |  |  |  |  |  |
| Mean | 87.30 | 104.2 | 95.90 | 86.40 | 95.30 | 94.80 | 79.20 | 94.90 | 103.5 |
| Std deviation | 31.50 | 44.07 | 24.34 | 27.57 | 29.42 | 25.11 | 17.72 | 28.18 | 29.57 |
| Std error of mean | 9.962 | 13.93 | 7.696 | 8.719 | 9.302 | 7.941 | 5.603 | 8.911 | 9.351 |
|  |  |  |  |  |  |  |  |  |  |
| Lower 95% CI | 64.76 | 72.68 | 78.49 | 66.68 | 74.26 | 76.84 | 66.52 | 74.74 | 82.33 |
| Upper 95% CI | 109.80 | 135.7 | 113.3 | 106.1 | 116.3 | 112.8 | 91.88 | 115.1 | 124.6 |

| **Albumin** | Carprofeno | |  | Meloxicam | |  | Paracetamol | |  |
| --- | --- | --- | --- | --- | --- | --- | --- | --- | --- |
|  | T0 | T48 | T96 | T0 | T48 | T96 | T0 | T48 | T96 |
|  | 3.6 | 3 | 3 | 4 | 3 | 3 | 3 | 4 | 4 |
|  | 4 | 4 | 4 | 4 | 3.2 | 4 | 3 | 3 | 3 |
|  | 4 | 4 | 4 | 4 | 4.2 | 4 | 3 | 3 | 3.4 |
|  | 3 | 3 | 3 | 3 | 3 | 3 | 3 | 3 | 3 |
|  | 3.4 | 3 | 3 | 3 | 3 | 3 | 4 | 3 | 3.4 |
|  | 3 | 3 | 3 | 3 | 3 | 3 | 4 | 4 | 3 |
|  | 3 | 3 | 4 | 3.4 | 3 | 3 | 4 | 3 | 3 |
|  | 3 | 4 | 3 | 3 | 3 | 3 | 3 | 6 | 3 |
|  | 3 | 3 | 3 | 3 | 3 | 3 | 3 | 3 | 3 |
|  | 3 | 3 | 3 | 4 | 4 | 4 | 3 | 3 | 3 |

**Albumin**

| Descriptive statistics | Carprofeno | | | Meloxicam | | | Paracetamol | | |
| --- | --- | --- | --- | --- | --- | --- | --- | --- | --- |
|  | T0 | T48 | T96 | T0 | T48 | T96 | T0 | T48 | T96 |
| Number of values | 10 | 10 | 10 | 10 | 10 | 10 | 10 | 10 | 10 |
|  |  |  |  |  |  |  |  |  |  |
| Minimum | 3 | 3 | 3 | 3 | 3 | 3 | 3 | 3 | 3 |
| 25% percentile | 3 | 3 | 3 | 3 | 3 | 3 | 3 | 3 | 3 |
| Median | 3 | 3 | 3 | 3.2 | 3 | 3 | 3 | 3 | 3 |
| 75% percentile | 3.7 | 4 | 4 | 4 | 3.4 | 4 | 4 | 4 | 3.4 |
| Maximum | 4 | 4 | 4 | 4 | 4.2 | 4 | 4 | 6 | 4 |
|  |  |  |  |  |  |  |  |  |  |
| Mean | 3.3 | 3.3 | 3.3 | 3.440 | 3.240 | 3.3 | 3.3 | 3.5 | 3.18 |
| Std deviation | 0.4243 | 0.4830 | 0.4830 | 0.4971 | 0.4600 | 0.4830 | 0.4830 | 0.9718 | 0.3327 |
| Std error of mean | 0.1342 | 0.1528 | 0.1528 | 0.1572 | 0.1454 | 0.1528 | 0.1528 | 0.3073 | 0.1052 |
|  |  |  |  |  |  |  |  |  |  |
| Lower 95% CI | 2.996 | 2.954 | 2.954 | 3.084 | 2.911 | 2.954 | 2.954 | 2.805 | 2.942 |
| Upper 95% CI | 3.604 | 3.646 | 3.646 | 3.796 | 3.569 | 3.646 | 3.646 | 4.195 | 3.418 |

| **Glucose** | Carprofeno | |  | Meloxicam | |  | Paracetamol | |  |
| --- | --- | --- | --- | --- | --- | --- | --- | --- | --- |
|  | T0 | T48 | T96 | T0 | T48 | T96 | T0 | T48 | T96 |
|  | 65 | 89 | 90 | 98 | 72 | 85 | 85 | 89 | 90 |
|  | 165 | 105 | 99 | 100 | 86 | 96 | 76 | 90 | 100 |
|  | 167 | 180 | 160 | 68 | 72 | 80 | 87 | 78 | 77 |
|  | 78 | 78 | 85 | 80 | 76 | 90 | 106 | 70 | 85 |
|  | 67 | 71 | 75 | 76 | 80 | 86 | 87 | 110 | 84 |
|  | 88 | 60 | 80 | 74 | 66 | 66 | 84 | 65 | 78 |
|  | 86 | 110 | 114 | 87 | 90 | 90 | 69 | 60 | 98 |
|  | 75 | 98 | 90 | 87 | 95 | 95 | 86 | 98 | 76 |
|  | 99 | 105 | 105 | 90 | 96 | 96 | 76 | 109 | 110 |
|  | 86 | 90 | 90 | 87 | 96 | 96 | 78 | 70 | 86 |

**Glucose**

| Descriptive statistics | Carprofeno | | | Meloxicam | | | Paracetamol | | |
| --- | --- | --- | --- | --- | --- | --- | --- | --- | --- |
|  | T0 | T48 | T96 | T0 | T48 | T96 | T0 | T48 | T96 |
| Number of values | 10 | 10 | 10 | 10 | 10 | 10 | 10 | 10 | 10 |
|  |  |  |  |  |  |  |  |  |  |
| Minimum | 65 | 60 | 75 | 68 | 66 | 66 | 69 | 60 | 76 |
| 25% percentile | 73 | 76.25 | 83.75 | 75.50 | 72 | 83.75 | 76 | 68.75 | 77.75 |
| Median | 86 | 94 | 90 | 87 | 83 | 90 | 84.50 | 83.50 | 85.50 |
| 75% percentile | 115.5 | 106.3 | 107.3 | 92 | 95.25 | 96 | 87 | 100.8 | 98.50 |
| Maximum | 167 | 180 | 160 | 100 | 96 | 96 | 106 | 110 | 110 |
|  |  |  |  |  |  |  |  |  |  |
| Mean | 97.60 | 98.60 | 98.80 | 84.70 | 82.90 | 88 | 83.40 | 83.90 | 88.40 |
| Std deviation | 37.44 | 32.80 | 24.40 | 10.25 | 11.20 | 9.487 | 9.958 | 18.02 | 11.18 |
| Std error of mean | 11.84 | 10.37 | 7.715 | 3.242 | 3.542 | 3.0 | 3.149 | 5.699 | 3.535 |
|  |  |  |  |  |  |  |  |  |  |
| Lower 95% CI | 70.82 | 75.14 | 81.35 | 77.37 | 74.89 | 81.21 | 76.28 | 71.01 | 80.40 |
| Upper 95% CI | 124.4 | 122.1 | 116.3 | 92.03 | 90.91 | 94.79 | 90.52 | 96.79 | 96.40 |

| **Urea** | Carprofeno | |  | Meloxicam | |  | Paracetamol | |  |
| --- | --- | --- | --- | --- | --- | --- | --- | --- | --- |
|  | T0 | T48 | T96 | T0 | T48 | T96 | T0 | T48 | T96 |
|  | 31 | 38.5 | 37 | 34.5 | 37.5 | 37.8 | 39.5 | 39 | 41 |
|  | 34.5 | 37.6 | 36.8 | 39.6 | 40.2 | 39.9 | 32.5 | 41.5 | 40.5 |
|  | 28 | 32 | 33 | 24.5 | 32.5 | 32 | 28.5 | 39.5 | 28.3 |
|  | 29.2 | 28.4 | 30 | 29.5 | 27 | 28.5 | 37.2 | 38.5 | 38 |
|  | 24.5 | 28 | 29 | 27.5 | 26.5 | 29 | 34.5 | 35.5 | 35.5 |
|  | 33 | 34.5 | 34.5 | 36.5 | 37.5 | 37.5 | 32.5 | 30.5 | 34.5 |
|  | 37.5 | 37 | 37 | 34.5 | 36.5 | 36.5 | 30.5 | 28.5 | 32.4 |
|  | 32.4 | 38.5 | 39.5 | 36.5 | 36.7 | 36.7 | 34.5 | 32.5 | 37.5 |
|  | 32 | 36.4 | 36.4 | 38.7 | 38 | 38 | 35.5 | 37.5 | 36.5 |
|  | 29.4 | 32.4 | 32.4 | 34.5 | 39.5 | 39.5 | 30.2 | 36.2 | 39.5 |

**Urea**

| Descriptive statistics | Carprofeno | | | Meloxicam | | | Paracetamol | | |
| --- | --- | --- | --- | --- | --- | --- | --- | --- | --- |
|  | T0 | T48 | T96 | T0 | T48 | T96 | T0 | T48 | T96 |
| Number of values | 10 | 10 | 10 | 10 | 10 | 10 | 10 | 10 | 10 |
|  |  |  |  |  |  |  |  |  |  |
| Minimum | 24.50 | 28 | 29 | 24.50 | 26.50 | 28.50 | 28.50 | 28.50 | 28.30 |
| 25% percentile | 28.90 | 31.10 | 31.80 | 29 | 31.13 | 31.25 | 30.43 | 32 | 33.98 |
| Median | 31.50 | 35.45 | 35.45 | 34.50 | 37.10 | 37.10 | 33.50 | 36.85 | 37 |
| 75% percentile | 33.38 | 37.83 | 37 | 37.05 | 38.38 | 38.38 | 35.93 | 39.13 | 39.75 |
| Maximum | 37.50 | 38.50 | 39.50 | 39.60 | 40.20 | 39.90 | 39.50 | 41.50 | 41 |
|  |  |  |  |  |  |  |  |  |  |
| Mean | 31.15 | 34.33 | 34.56 | 33.63 | 35.19 | 35.54 | 33.54 | 35.92 | 36.37 |
| Std deviation | 3.625 | 3.962 | 3.392 | 4.923 | 4.90 | 4.174 | 3.376 | 4.203 | 3.907 |
| Std error of mean | 1.146 | 1.253 | 1.073 | 1.557 | 1.549 | 1.320 | 1.068 | 1.329 | 1.235 |
|  |  |  |  |  |  |  |  |  |  |
| Lower 95% CI | 28.56 | 31.50 | 32.13 | 30.11 | 31.68 | 32.55 | 31.13 | 32.91 | 33.58 |
| Upper 95% CI | 33.74 | 37.16 | 36.99 | 37.15 | 38.70 | 38.53 | 35.95 | 38.93 | 39.16 |

| **Creatinine** | Carprofeno | |  | Meloxicam | |  | Paracetamol | |  |
| --- | --- | --- | --- | --- | --- | --- | --- | --- | --- |
|  | T0 | T48 | T96 | T0 | T48 | T96 | T0 | T48 | T96 |
|  | 0.9 | 0.8 | 0.8 | 0.4 | 0.7 | 0.8 | 0.8 | 0.9 | 0.9 |
|  | 0.7 | 0.8 | 0.8 | 0.9 | 0.8 | 0.8 | 0.4 | 1.1 | 1 |
|  | 0.4 | 0.7 | 0.8 | 0.5 | 0.6 | 0.7 | 0.7 | 0.9 | 0.6 |
|  | 0.6 | 0.7 | 0.7 | 0.7 | 0.6 | 0.7 | 0.9 | 0.8 | 0.6 |
|  | 0.5 | 0.4 | 0.5 | 0.6 | 0.7 | 0.6 | 0.6 | 0.4 | 0.4 |
|  | 0.6 | 0.5 | 0.5 | 0.8 | 0.8 | 0.8 | 0.4 | 0.5 | 0.7 |
|  | 0.5 | 0.7 | 0.8 | 0.6 | 0.7 | 0.7 | 0.4 | 0.3 | 0.6 |
|  | 0.4 | 0.7 | 0.6 | 0.6 | 0.7 | 0.7 | 0.6 | 0.5 | 0.6 |
|  | 0.4 | 0.5 | 0.5 | 0.7 | 0.7 | 0.7 | 0.6 | 0.7 | 0.8 |
|  | 0.6 | 0.7 | 0.7 | 0.6 | 1.2 | 1.2 | 0.4 | 0.6 | 0.6 |

**Creatinine**

| Descriptive statistics | Carprofeno | | | Meloxicam | | | Paracetamol | | |
| --- | --- | --- | --- | --- | --- | --- | --- | --- | --- |
|  | T0 | T48 | T96 | T0 | T48 | T96 | T0 | T48 | T96 |
| Number of values | 10 | 10 | 10 | 10 | 10 | 10 | 10 | 10 | 10 |
|  |  |  |  |  |  |  |  |  |  |
| Minimum | 0.4 | 0.4 | 0.5 | 0.4 | 0.6 | 0.6 | 0.4 | 0.3 | 0.4 |
| 25% percentile | 0.4 | 0.5 | 0.5 | 0.575 | 0.675 | 0.7 | 0.4 | 0.475 | 0.6 |
| Median | 0.55 | 0.7 | 0.7 | 0.6 | 0.7 | 0.7 | 0.6 | 0.65 | 0.6 |
| 75% percentile | 0.625 | 0.725 | 0.8 | 0.725 | 0.8 | 0.8 | 0.725 | 0.9 | 0.825 |
| Maximum | 0.9 | 0.8 | 0.8 | 0.9 | 1.2 | 1.2 | 0.9 | 1.1 | 1.0 |
|  |  |  |  |  |  |  |  |  |  |
| Mean | 0.56 | 0.65 | 0.67 | 0.64 | 0.75 | 0.77 | 0.58 | 0.67 | 0.68 |
| Std deviation | 0.1578 | 0.1354 | 0.1337 | 0.1430 | 0.1716 | 0.1636 | 0.1814 | 0.2541 | 0.1751 |
| Std error of mean | 0.04989 | 0.04282 | 0.04230 | 0.04522 | 0.05426 | 0.05175 | 0.05735 | 0.08035 | 0.05538 |
|  |  |  |  |  |  |  |  |  |  |
| Lower 95% CI | 0.4471 | 0.5531 | 0.5743 | 0.5377 | 0.6272 | 0.6529 | 0.4503 | 0.4882 | 0.5547 |
| Upper 95% CI | 0.6729 | 0.7469 | 0.7657 | 0.7423 | 0.8728 | 0.8871 | 0.7097 | 0.8518 | 0.8053 |

| **Total bilirubin** | Carprofeno | |  | Meloxicam | |  | Paracetamol | |  |
| --- | --- | --- | --- | --- | --- | --- | --- | --- | --- |
|  | T0 | T48 | T96 | T0 | T48 | T96 | T0 | T48 | T96 |
|  | 0.4 | 0.5 | 0.4 | 0.4 | 0.5 | 0.6 | 0.3 | 0.5 | 0.5 |
|  | 0.3 | 0.3 | 0.4 | 0.4 | 0.7 | 0.5 | 0.2 | 0.4 | 0.4 |
|  | 0.6 | 0.7 | 0.6 | 0.4 | 0.4 | 0.4 | 0.5 | 0.5 | 0.5 |
|  | 0.6 | 0.5 | 0.6 | 0.3 | 0.3 | 0.4 | 0.5 | 0.4 | 0.3 |
|  | 0.4 | 0.5 | 0.5 | 0.5 | 0.4 | 0.4 | 0.2 | 0.3 | 0.4 |
|  | 0.2 | 0.4 | 0.4 | 0.3 | 0.3 | 0.3 | 0.5 | 0.4 | 0.5 |
|  | 0.3 | 0.6 | 0.7 | 0.4 | 0.5 | 0.5 | 0.5 | 0.7 | 0.9 |
|  | 0.2 | 0.4 | 0.4 | 0.5 | 0.7 | 0.7 | 0.3 | 0.4 | 0.4 |
|  | 0.5 | 0.5 | 0.6 | 0.5 | 0.5 | 0.5 | 0.5 | 0.7 | 0.9 |
|  | 0.4 | 0.5 | 0.5 | 0.4 | 0.4 | 0.4 | 0.3 | 0.6 | 0.5 |

**Total bilirubin**

| Descriptive statistics | Carprofeno | | | Meloxicam | | | Paracetamol | | |
| --- | --- | --- | --- | --- | --- | --- | --- | --- | --- |
|  | T0 | T48 | T96 | T0 | T48 | T96 | T0 | T48 | T96 |
| Number of values | 10 | 10 | 10 | 10 | 10 | 10 | 10 | 10 | 10 |
|  |  |  |  |  |  |  |  |  |  |
| Minimum | 0.2 | 0.3 | 0.4 | 0.3 | 0.3 | 0.3 | 0.2 | 0.3 | 0.3 |
| 25% percentile | 0.275 | 0.4 | 0.4 | 0.375 | 0.375 | 0.4 | 0.275 | 0.4 | 0.4 |
| Median | 0.4 | 0.5 | 0.5 | 0.4 | 0.45 | 0.45 | 0.4 | 0.45 | 0.5 |
| 75% percentile | 0.525 | 0.525 | 0.6 | 0.5 | 0.55 | 0.525 | 0.5 | 0.625 | 0.6 |
| Maximum | 0.6 | 0.7 | 0.7 | 0.5 | 0.7 | 0.7 | 0.5 | 0.7 | 0.9 |
|  |  |  |  |  |  |  |  |  |  |
| Mean | 0.39 | 0.49 | 0.51 | 0.41 | 0.47 | 0.47 | 0.38 | 0.49 | 0.53 |
| Std deviation | 0.1449 | 0.1101 | 0.1101 | 0.07379 | 0.1418 | 0.1160 | 0.1317 | 0.1370 | 0.2058 |
| Std error of mean | 0.04583 | 0.03480 | 0.03480 | 0.02333 | 0.04485 | 0.03667 | 0.04163 | 0.04333 | 0.06506 |
|  |  |  |  |  |  |  |  |  |  |
| Lower 95% CI | 0.2863 | 0.4113 | 0.4313 | 0.3572 | 0.3686 | 0.3871 | 0.2858 | 0.3920 | 0.3828 |
| Upper 95% CI | 0.4937 | 0.5687 | 0.5887 | 0.4628 | 0.5714 | 0.5529 | 0.4742 | 0.5880 | 0.6772 |

| **Direct bilirubin** | Carprofeno | |  | Meloxicam | |  | Paracetamol | |  |
| --- | --- | --- | --- | --- | --- | --- | --- | --- | --- |
|  | T0 | T48 | T96 | T0 | T48 | T96 | T0 | T48 | T96 |
|  | 0.3 | 0.3 | 0.3 | 0.3 | 0.3 | 0.4 | 0.2 | 0.4 | 0.4 |
|  | 0.2 | 0.2 | 0.3 | 0.3 | 0.5 | 0.4 | 0.1 | 0.3 | 0.3 |
|  | 0.4 | 0.5 | 0.4 | 0.3 | 0.3 | 0.3 | 0.3 | 0.3 | 0.3 |
|  | 0.4 | 0.3 | 0.4 | 0.3 | 0.2 | 0.3 | 0.4 | 0.3 | 0.2 |
|  | 0.3 | 0.3 | 0.4 | 0.3 | 0.3 | 0.3 | 0.2 | 0.2 | 0.3 |
|  | 0.2 | 0.3 | 0.3 | 0.2 | 0.2 | 0.2 | 0.3 | 0.3 | 0.3 |
|  | 0.2 | 0.4 | 0.6 | 0.3 | 0.3 | 0.3 | 0.3 | 0.5 | 0.6 |
|  | 0.1 | 0.3 | 0.3 | 0.3 | 0.5 | 0.5 | 0.2 | 0.3 | 0.3 |
|  | 0.3 | 0.3 | 0.4 | 0.3 | 0.3 | 0.3 | 0.3 | 0.5 | 0.6 |
|  | 0.3 | 0.3 | 0.3 | 0.3 | 0.3 | 0.3 | 0.2 | 0.4 | 0.4 |

**Direct bilirubin**

| Descriptive statistics | Carprofeno | | | Meloxicam | | | Paracetamol | | |
| --- | --- | --- | --- | --- | --- | --- | --- | --- | --- |
|  | T0 | T48 | T96 | T0 | T48 | T96 | T0 | T48 | T96 |
| Number of values | 10 | 10 | 10 | 10 | 10 | 10 | 10 | 10 | 10 |
|  |  |  |  |  |  |  |  |  |  |
| Minimum | 0.1 | 0.2 | 0.3 | 0.2 | 0.2 | 0.2 | 0.1 | 0.2 | 0.2 |
| 25% percentile | 0.2 | 0.3 | 0.3 | 0.3 | 0.275 | 0.3 | 0.2 | 0.3 | 0.3 |
| Median | 0.3 | 0.3 | 0.35 | 0.3 | 0.3 | 0.3 | 0.25 | 0.3 | 0.3 |
| 75% percentile | 0.325 | 0.325 | 0.4 | 0.3 | 0.35 | 0.4 | 0.3 | 0.425 | 0.45 |
| Maximum | 0.4 | 0.5 | 0.6 | 0.3 | 0.5 | 0.5 | 0.4 | 0.5 | 0.6 |
|  |  |  |  |  |  |  |  |  |  |
| Mean | 0.27 | 0.32 | 0.37 | 0.29 | 0.32 | 0.33 | 0.25 | 0.35 | 0.37 |
| Std deviation | 0.09487 | 0.07888 | 0.09487 | 0.03162 | 0.1033 | 0.08233 | 0.08498 | 0.09718 | 0.1337 |
| Std error of mean | 0.03 | 0.02494 | 0.03 | 0.01 | 0.03266 | 0.02603 | 0.02687 | 0.03073 | 0.04230 |
|  |  |  |  |  |  |  |  |  |  |
| Lower 95% CI | 0.2021 | 0.2636 | 0.3021 | 0.2674 | 0.2461 | 0.2711 | 0.1892 | 0.2805 | 0.2743 |
| Upper 95% CI | 0.3379 | 0.3764 | 0.4379 | 0.3126 | 0.3939 | 0.3889 | 0.3108 | 0.4195 | 0.4657 |

| **Indirect bilirubin** | Carprofeno | |  | Meloxicam | |  | Paracetamol | |  |
| --- | --- | --- | --- | --- | --- | --- | --- | --- | --- |
|  | T0 | T48 | T96 | T0 | T48 | T96 | T0 | T48 | T96 |
|  | 0.1 | 0.2 | 0.1 | 0.1 | 0.2 | 0.2 | 0.1 | 0.1 | 0.1 |
|  | 0.1 | 0.1 | 0.1 | 0.1 | 0.2 | 0.1 | 0.1 | 0.1 | 0.1 |
|  | 0.2 | 0.2 | 0.2 | 0.1 | 0.1 | 0.1 | 0.2 | 0.2 | 0.2 |
|  | 0.2 | 0.2 | 0.2 | 0 | 0.1 | 0.1 | 0.1 | 0.1 | 0.1 |
|  | 0.1 | 0.2 | 0.1 | 0.2 | 0.1 | 0.1 | 0 | 0.1 | 0.1 |
|  | 0 | 0.1 | 0.1 | 0.1 | 0.1 | 0.1 | 0.2 | 0.1 | 0.2 |
|  | 0.1 | 0.2 | 0.1 | 0.1 | 0.2 | 0.2 | 0.2 | 0.2 | 0.3 |
|  | 0.1 | 0.1 | 0.1 | 0.2 | 0.2 | 0.2 | 0.1 | 0.1 | 0.1 |
|  | 0.2 | 0.2 | 0.2 | 0.2 | 0.2 | 0.2 | 0.2 | 0.2 | 0.3 |
|  | 0.1 | 0.2 | 0.2 | 0.1 | 0.1 | 0.1 | 0.1 | 0.2 | 0.1 |

**Indirect bilirubin**

| Descriptive statistics | Carprofeno | | | Meloxicam | | | Paracetamol | | |
| --- | --- | --- | --- | --- | --- | --- | --- | --- | --- |
|  | T0 | T48 | T96 | T0 | T48 | T96 | T0 | T48 | T96 |
| Number of values | 10 | 10 | 10 | 10 | 10 | 10 | 10 | 10 | 10 |
|  |  |  |  |  |  |  |  |  |  |
| Minimum | 0.0 | 0.1 | 0.1 | 0.0 | 0.1 | 0.1 | 0.0 | 0.1 | 0.1 |
| 25% percentile | 0.1 | 0.1 | 0.1 | 0.1 | 0.1 | 0.1 | 0.1 | 0.1 | 0.1 |
| Median | 0.1 | 0.2 | 0.1 | 0.1 | 0.15 | 0.1 | 0.1 | 0.1 | 0.1 |
| 75% percentile | 0.2 | 0.2 | 0.2 | 0.2 | 0.2 | 0.2 | 0.2 | 0.2 | 0.225 |
| Maximum | 0.2 | 0.2 | 0.2 | 0.2 | 0.2 | 0.2 | 0.2 | 0.2 | 0.3 |
|  |  |  |  |  |  |  |  |  |  |
| Mean | 0.12 | 0.17 | 0.14 | 0.12 | 0.15 | 0.14 | 0.13 | 0.14 | 0.16 |
| Std deviation | 0.06325 | 0.04830 | 0.05164 | 0.06325 | 0.05270 | 0.05164 | 0.06749 | 0.05164 | 0.08433 |
| Std error of mean | 0.02 | 0.01528 | 0.01633 | 0.02 | 0.01667 | 0.01633 | 0.2134 | 0.01633 | 0.02667 |
|  |  |  |  |  |  |  |  |  |  |
| Lower 95% CI | 0.07476 | 0.1354 | 0.1031 | 0.07476 | 0.1123 | 0.1031 | 0.08172 | 0.1031 | 0. 0996 |
| Upper 95% CI | 0.1652 | 0.2046 | 0.1769 | 0.1652 | 0.1877 | 0.1769 | 0.1783 | 0.1769 | 0.2203 |
