# Supplementary material for: Clinical evaluation of postoperative analgesia, cardiorespiratory parameters and changes in liver and renal function tests of paracetamol compared to meloxicam and carprofen in dogs undergoing ovariohysterectomy
Source: PLoS One. 2020 Feb 14;15(2):e0223697. doi: 10.1371/journal.pone.0223697 (PMC7021320; doi:10.1371/journal.pone.0223697)
Supplement: S1 Fig — (DOCX) [file pone.0223697.s004.docx]

| DIVAS |  |  |  |  |  |  |  |  |  |  |  |  |  |  |  |  |  |  |  |  |  |  |  |  |  |  |  |  |  |  |  |  |
| --- | --- | --- | --- | --- | --- | --- | --- | --- | --- | --- | --- | --- | --- | --- | --- | --- | --- | --- | --- | --- | --- | --- | --- | --- | --- | --- | --- | --- | --- | --- | --- | --- |
| Carp 1h | Melox 1h | Para 1h | Carp 2h | Melox 2h | Para 2h | Carp 4h | Melox 4h | Para 4h | Carp 6h | Melox 6h | Para 6h | Carp 8h | Melox 8h | Para 8h | Carp 12h | Melox 12h | Para 12h | Carp 16h | Melox 16h | Para 16h | Carp 20h | Melox 20h | Para 20h | Carp 24h | Melox 24h | Para 24h | Carp 36h | Melox 36h | Para 36h | Carp 48h | Melox 48h | Para 48h |
| 40 | 40 | 30 | 10 | 30 | 30 | 20 | 10 | 20 | 0 | 10 | 20 | 0 | 10 | 10 | 10 | 0 | 10 | 10 | 0 | 10 | 10 | 0 | 10 | 10 | 0 | 10 | 0 | 0 | 0 | 0 | 0 | 0 |
| 20 | 30 | 30 | 20 | 20 | 20 | 20 | 20 | 20 | 10 | 10 | 10 | 10 | 10 | 10 | 10 | 10 | 10 | 0 | 10 | 10 | 0 | 0 | 10 | 0 | 0 | 10 | 0 | 0 | 10 | 0 | 0 | 10 |
| 30 | 30 | 20 | 30 | 20 | 10 | 10 | 20 | 10 | 10 | 10 | 0 | 10 | 10 | 0 | 0 | 10 | 0 | 0 | 10 | 10 | 0 | 10 | 10 | 0 | 0 | 0 | 0 | 0 | 0 | 0 | 0 | 0 |
| 40 | 20 | 20 | 50 | 20 | 20 | 20 | 20 | 20 | 10 | 20 | 20 | 10 | 0 | 10 | 0 | 0 | 10 | 0 | 0 | 10 | 0 | 0 | 10 | 0 | 0 | 0 | 0 | 0 | 0 | 0 | 0 | 0 |
| 20 | 10 | 20 | 10 | 10 | 20 | 10 | 10 | 10 | 0 | 0 | 10 | 0 | 0 | 10 | 0 | 0 | 10 | 0 | 0 | 0 | 0 | 0 | 0 | 0 | 0 | 0 | 0 | 0 | 0 | 0 | 0 | 0 |
| 10 | 20 | 20 | 10 | 20 | 10 | 10 | 10 | 10 | 10 | 10 | 0 | 10 | 0 | 0 | 10 | 0 | 0 | 0 | 0 | 0 | 0 | 0 | 0 | 0 | 0 | 0 | 0 | 0 | 0 | 0 | 0 | 0 |
| 30 | 30 | 20 | 20 | 30 | 20 | 10 | 20 | 10 | 10 | 20 | 10 | 10 | 10 | 0 | 0 | 10 | 0 | 0 | 0 | 0 | 0 | 0 | 0 | 0 | 0 | 0 | 0 | 0 | 0 | 0 | 0 | 0 |
| 20 | 20 | 20 | 20 | 20 | 20 | 20 | 10 | 20 | 10 | 0 | 10 | 10 | 0 | 10 | 10 | 0 | 10 | 0 | 0 | 10 | 0 | 0 | 0 | 0 | 0 | 0 | 0 | 0 | 0 | 0 | 0 | 0 |
| 10 | 10 | 30 | 10 | 10 | 20 | 0 | 10 | 20 | 0 | 0 | 10 | 0 | 0 | 10 | 0 | 0 | 10 | 0 | 0 | 10 | 0 | 0 | 0 | 0 | 0 | 0 | 0 | 0 | 0 | 0 | 0 | 0 |
| 20 | 20 | 20 | 10 | 10 | 10 | 10 | 10 | 10 | 10 | 0 | 10 | 10 | 0 | 10 | 10 | 0 | 10 | 0 | 0 | 10 | 0 | 0 | 10 | 0 | 0 | 0 | 0 | 0 | 0 | 0 | 0 | 0 |

S1 Figure 1. Scores obtained on the DIVAS scale to determine the degree of pain in each of the individuals of the different study groups for 48 hours postoperatively after performing an elective ovariohysterectomy.

Descriptive statistics

|  | Carp 1h | Melox 1h | Para 1h | Carp 2h | Melox 2h | Para 2h | Carp 4h | Melox 4h | Para 4h | Carp 6h | Melox 6h | Para 6h | Carp 8h | Melox 8h | Para 8h | Carp 12h | Melox 12h | Para 12h | Carp 16h | Melox 16h | Para 16h | Carp 20h | Melox 20h | Para 20h | Carp 24h | Melox 24h | Para 24h | Carp 36h | Melox 36h | Para 36h | Carp 48h | Melox 48h | Para 48h |
| --- | --- | --- | --- | --- | --- | --- | --- | --- | --- | --- | --- | --- | --- | --- | --- | --- | --- | --- | --- | --- | --- | --- | --- | --- | --- | --- | --- | --- | --- | --- | --- | --- | --- |
| Number of values | 10 | 10 | 10 | 10 | 10 | 10 | 10 | 10 | 10 | 10 | 10 | 10 | 10 | 10 | 10 | 10 | 10 | 10 | 10 | 10 | 10 | 10 | 10 | 10 | 10 | 10 | 10 | 10 | 10 | 10 | 10 | 10 | 10 |
|  |  |  |  |  |  |  |  |  |  |  |  |  |  |  |  |  |  |  |  |  |  |  |  |  |  |  |  |  |  |  |  |  |  |
| Minimum | 10 | 10 | 20 | 10 | 10 | 10 | 0 | 10 | 10 | 0 | 0 | 0 | 0 | 0 | 0 | 0 | 0 | 0 | 0 | 0 | 0 | 0 | 0 | 0 | 0 | 0 | 0 | 0 | 0 | 0 | 0 | 0 | 0 |
| 25% percentile | 17.5 | 17.5 | 20 | 10 | 10 | 10 | 10 | 10 | 10 | 0 | 0 | 7.5 | 0 | 0 | 0 | 0 | 0 | 0 | 0 | 0 | 0 | 0 | 0 | 0 | 0 | 0 | 0 | 0 | 0 | 0 | 0 | 0 | 0 |
| Median | 20 | 20 | 20 | 15 | 20 | 20 | 10 | 10 | 15 | 10 | 10 | 10 | 10 | 0 | 10 | 5 | 0 | 10 | 0 | 0 | 10 | 0 | 0 | 5 | 0 | 0 | 0 | 0 | 0 | 0 | 0 | 0 | 0 |
| 75% percentile | 32.5 | 30 | 30 | 22.5 | 22.5 | 20 | 20 | 20 | 20 | 10 | 12.5 | 12.5 | 10 | 10 | 10 | 10 | 10 | 10 | 0 | 2.5 | 10 | 0 | 0 | 10 | 0 | 0 | 2.5 | 0 | 0 | 0 | 0 | 0 | 0 |
| Maximum | 40 | 40 | 30 | 50 | 30 | 30 | 20 | 20 | 20 | 10 | 20 | 20 | 10 | 10 | 10 | 10 | 10 | 10 | 10 | 10 | 10 | 10 | 10 | 10 | 10 | 0 | 10 | 0 | 0 | 10 | 0 | 0 | 10 |
|  |  |  |  |  |  |  |  |  |  |  |  |  |  |  |  |  |  |  |  |  |  |  |  |  |  |  |  |  |  |  |  |  |  |
| Mean | 24 | 23 | 23 | 19 | 19 | 18 | 13 | 14 | 15 | 7 | 8 | 10 | 7 | 4 | 7 | 5 | 3 | 7 | 1 | 2 | 7 | 1 | 1 | 5 | 1 | 0 | 2 | 0 | 0 | 1 | 0 | 0 | 1 |
| Std deviation | 10.75 | 9.48 | 4.83 | 12.87 | 7.37 | 6.32 | 6.74 | 5.16 | 5.27 | 4.83 | 7.88 | 6.66 | 4.83 | 5.16 | 4.83 | 5.2 | 4.8 | 4.8 | 3.16 | 4.21 | 4.83 | 3.16 | 3.16 | 5.27 | 3.16 | 0 | 4.21 | 0 | 0 | 3.16 | 0 | 0 | 3.16 |
| Std error of mean | 3.399 | 3.0 | 1.52 | 4.07 | 2.33 | 2.0 | 2.13 | 1.63 | 1.66 | 1.52 | 2.49 | 2.1 | 1.52 | 1.63 | 1.52 | 1.66 | 1.52 | 1.52 | 1 | 1.33 | 1.52 | 1 | 1 | 1 | 1 | 0 | 1.33 | 0 | 0 | 1 | 0 | 0 | 1 |
|  |  |  |  |  |  |  |  |  |  |  |  |  |  |  |  |  |  |  |  |  |  |  |  |  |  |  |  |  |  |  |  |  |  |
| Lower 95% CI | 16.31 | 16.21 | 19.54 | 9.79 | 13.7 | 13.5 | 8.17 | 10.3 | 11.2 | 3.54 | 2.35 | 5.23 | 3.54 | 0.3 | 3.54 | 1.23 | -0.45 | 3.54 | -1.26 | -1.01 | 3.54 | -1.26 | -1.26 | 1.23 | -1.26 | 0 | -1.01 | 0 | 0 | -1.26 | 0 | 0 | -1.26 |
| Upper 95% CI | 31.69 | 29.79 | 26.46 | 28.2 | 24.3 | 22.5 | 17.8 | 17.7 | 18.7 | 10.4 | 13.6 | 14.7 | 10.4 | 7.7 | 10.4 | 8.77 | 6.45 | 10.4 | 3.26 | 5.01 | 10.46 | 3.26 | 3.26 | 8.77 | 3.26 | 0 | 5.01 | 0 | 0 | 3.26 | 0 | 0 | 3.26 |
|  |  |  |  |  |  |  |  |  |  |  |  |  |  |  |  |  |  |  |  |  |  |  |  |  |  |  |  |  |  |  |  |  |  |
| Mean ranks | 30.15 | 30.15 | 30.5 | 27.05 | 28.3 | 27.65 | 24.1 | 25.3 | 26.1 | 17.9 | 17.7 | 20.8 | 17.90 | 13.8 | 18.05 | 15.4 | 12.5 | 18.0 | 10 | 11.3 | 17.9 | 10 | 10.1 | 15.15 | 10 | 8.75 | 11.2 | 8.75 | 8.75 | 9.95 | 8.75 | 8.75 | 9.995 |
