# Supplementary material for: Clinical evaluation of postoperative analgesia, cardiorespiratory parameters and changes in liver and renal function tests of paracetamol compared to meloxicam and carprofen in dogs undergoing ovariohysterectomy
Source: PLoS One. 2020 Feb 14;15(2):e0223697. doi: 10.1371/journal.pone.0223697 (PMC7021320; doi:10.1371/journal.pone.0223697)
Supplement: Attachment — Submitted filename: reviewer response.docx [file pone.0223697.s006.docx]

Response to Academic Editor

 At this time, we request that you please report additional details in your Methods section regarding animal care, as per our editorial guidelines:

(1) Please state the breeds of the dogs used in this study

Thanks for your comment the observation has been placed in material and method

Line 124 – 125. (11 mixed breed, 7 poodle, 3 beagle, 2 schnauzer, 2 pug, 2 cocker spaniel, 1 doberman, 1 pit bull, 1 Australian shepherd).

(2) Please provide details of animal welfare (e.g., shelter, food, water, environmental enrichment)

Thanks for your comment the observation has been placed in material and method

. Line 129 – 135. Animals were received and placed in individual accommodation 24 hours before surgery with water and food *ad libitum*. The fasting time was 8 hours of solids and 2 hours of liquids.

(3) Please describe any steps taken to minimize animal suffering and distress, such as by administering anesthesia.

All animals had ownership and are accustomed to handling, catheterization is performed with minimal physical containment.

Subsequently the induction of anesthesia in order to avoid stress. At all times they were monitored (intraoperatively and postoperatively) in order to avoid pain and suffering.

Animals that were observed a score that indicated moderate pain were rescued with the administration of tramadol 2 mg kg intravenously

Thank you for your attention to these requests.

2. Thank you for providing a copy of the criteria used in the University of Melbourne's Pain Scale (UPMS). At this time, we also ask that you please provide a copy of the Dynamic Interactive Visual Analog Scale (DIVAS) scoring system if is not under a copyright more restrictive than CC-BY. Please include a copy, in both the original language and English, as Supporting Information to ensure that you have provided sufficient details that others could replicate the analyses.

Thank you for your comment, the DIVAS scale has been attached to Appendix 1 and the evaluation criteria is described.

3. Thank you for stating that consent was obtained from the owners of the female dogs used in this study. At this time, we also ask that you please provide additional details regarding owner consent. Please ensure that you have specified (1) whether consent was informed and (2) what type you obtained (for instance, written or verbal, and if verbal, how it was documented and witnessed).

Thank you for your comment; the type of consent has been placed.

Line 125-126: presented for elective ovariohysterectomy and were used in the present study after obtaining written informed owner consent.

4. Thank you for including your competing interests’ statement;

Please complete your Competing Interests on the online submission form to state any Competing Interests. If you have no competing interests, please state "The authors have declared that no competing interests exist.", as detailed online in our guide for authors at <http://journals.plos.org/plosone/s/submit-now>

 This information should be included in your cover letter; we will change the online submission form on your behalf.

Please update your statement in English

Please know it is PLOS ONE policy for corresponding authors to declare, on behalf of all authors, all potential competing interests for the purposes of transparency. PLOS defines a competing interest as anything that interferes with, or could reasonably be perceived as interfering with, the full and objective presentation, peer review, editorial decision-making, or publication of research or non-research articles submitted to one of the journals. Competing interests can be financial or non-financial, professional, or personal. Competing interests can arise in relationship to an organization or another person. Please follow this link to our website for more details on competing interests: <http://journals.plos.org/plosone/s/competing-interests>

Thank you for your comment

The following statement has been placed on the cover letter:

The authors have stated that there are no competing interests of any kind that interfere with the complete and objective presentation, peer review, editorial decision-making or the publication of research or non-research articles sent to PLOS One, whether financial or non-financial, professional or personal.

5. Please amend either the abstract on the online submission form (via Edit Submission) or the abstract in the manuscript so that they are identical.

Review Comments to the Author

Please use the space provided to explain your answers to the questions above. You may also include additional comments for the author, including concerns about dual publication, research ethics, or publication ethics. (Please upload your review as an attachment if it exceeds 20,000 characters)

Reviewer #1: Multimodal analgesia becoming very popular all for the purpose of decreasing pain and avoid the side effect of narcotics.
In conclusion there was no significant difference between the 3 drugs due limited number of the cases in the study and lack of serum concentration of the 3 different drugs.

Thank you for your comment

These observations have been included as limitations of the study in the final part of the discussion of this manuscript (Line 354-355)

Reviewer #2: This study is a pain study in female dogs who had hysterectomy and salpingopherectomy. The authors compared the analgesic effect of paracetamol, carprofen or meloxicam for postoperative pain management and biochemical changes.

It think it is better to replace the biochemical changes with liver and kidney function tests and the hemodynamics. Biochemical changes imply that the blood levels of medications will be measured.

Thank you for your comment, the title has been modified for a better understanding of the reader by:

Clinical evaluation of postoperative analgesia, cardiorespiratory parameters and changes in liver and renal function tests of paracetamol compared to meloxicam and carprofen in dogs undergoing ovariohysterectomy

Sample size calculation needs to be included. It is hard to draw conclusions from a study of 10 patients in each group.

Thank you for your comment, this section has been included in materials and methods taking as reference what was cited by Charan and Biswas, 2013, in relation to the determination of the sample size in clinical studies. Line 127-128.

I'm not sure why did the study look at the liver and kidney functions while using the therapeutic doses of the medications and expect to have a difference. The only difference that was reported was slight increase of the ALT on day 4.

Thanks for your comment, however, the use of paracetamol is still rare in dogs, although there are therapeutic doses on the use of paracetamol in dogs, it is also true that there are also several reports that document the liver toxicity of this drug even when therapeutic doses are used.

The study also, looked at the hemodynamic changes of medications that are known to not cause any significant changes. The only reported a significant change was a decrease of MAP in the carpoprofen group. They found the MAP in the carprofen group to be 71+/- 14 compared to 82+/- 17 and 84+/- 16 which is still in the acceptable range.

Thank you for your comment

It is correct all the mean blood pressure values in the different treatments were kept in normal ranges

The introduction and the discussion should be shortened.

Thank you for your comment

The introduction has been shortened
